# Supplementary material for: Downregulation of LncRNA GCLC-1 Promotes Microcystin-LR-Induced Malignant Transformation of Human Liver Cells by Regulating GCLC Expression
Source: Toxics. 2023 Feb 9;11(2):162. doi: 10.3390/toxics11020162 (PMC9960881; doi:10.3390/toxics11020162)
Supplement: Supplementary file 1 [file toxics-11-00162-s001.zip › toxics-2143435-supplementary.pdf]

Supplementary Materials: Downregulation of LncRNA GCLC-1 Promotes Microcystin-LR-Induced Malignant Transformation of Human Liver Cells by Regulating GCLC Expression

Xinglei Huang, Zhaohui Su, Jiangheng Li, Junquan He, Na Zhao, Liyun Nie, Bin Guan, Qiuyue Huang, Huiliu Zhao, Guo-Dong Lu and Qingqing Nong

Supplementary Table S1 Primer sequences used in the experiment.

Gene name	Forward primer (5′→3′)	Reverse primer (5′→3′)
lncGCLC	CCTACTTGTCTGGTGCCCAT	CCCCAGCTGTCAAGGAAAGG
GCLC	GGGGCGATGAGGTGGAAT	CCTTCAATCATGTAACTCCCATAC
ELOVL5	CAGCAGCTGCCAGAAAACAG	CAGGGGACCCAGTTCATCAC
TMEM14A	TTTGGTTATGCAGCCCTCGT	ATAGCCGGCCAAACATCCAA
ICK	AGCAGCCCAGATTCACCAAAA	CACCAGTCAGCTGAGGGAAA
FBXO9	CAGAGGGCAACTGGTGTGTT	GCCCCAAACTTTCAAGCAGG
miR-122-5p	AATCGGCGTGGAGTGTGACAAT	ATCCAGTGCAGGGTCCGAGG
GAPDH	CATGAGAAGTATGACAACAGCCT	AGTCCTTCCACGATACCAAAGT
U6	ACAGATCTGTCGGTGTGGCAC	GGCCCCGGATTATCCGACATTC

Supplementary Table S2 Clinicopathologic factors of hepatocellular carcinoma patients with MC exposure.

**Clinicopathologic factor**	**Low MCs exposure (n = 13) N (%)**	**High MCs exposure (n = 17) N (%)**	***P a***
Gender			
Male	8 (61.54)	13 (76.47)	0.443
Female	5 (38.46)	4 (23.53)	
Age (years)			
> 50	4 (30.77)	8 (47.06)	0.465
≤ 50	9 (69.23)	9 (52.94)	
HBV infection			
Yes	10 (76.93)	15 (88.24)	0.628
No	3 (23.07)	2 (11.76)	
Tumor size			
≥ 5 cm	6 (46.15)	10 (58.82)	0.713
< 5 cm	7 (53.85)	7 (41.18)	
Differentiation			
Well	4 (30.77)	7 (41.18)	0.708
Poor/moderate	9 (69.23)	10 (58.82)	
Lymph node metastasis			
Yes	8 (61.54)	15 (88.24)	0.101
No	5 (38.46)	2 (11.76)	
BCLC stages			
0 ~ A	3 (23.08)	6 (35.29)	0.471
B	4 (30.77)	7 (41.18)	
C ~ D	6 (46.15)	4 (23.53)	

Note: [a] *P* values for a two-sided χ^2 test. Abbreviations: MCs, Microcystins; BCLC, Barcelona Clinic Liver Cancer.

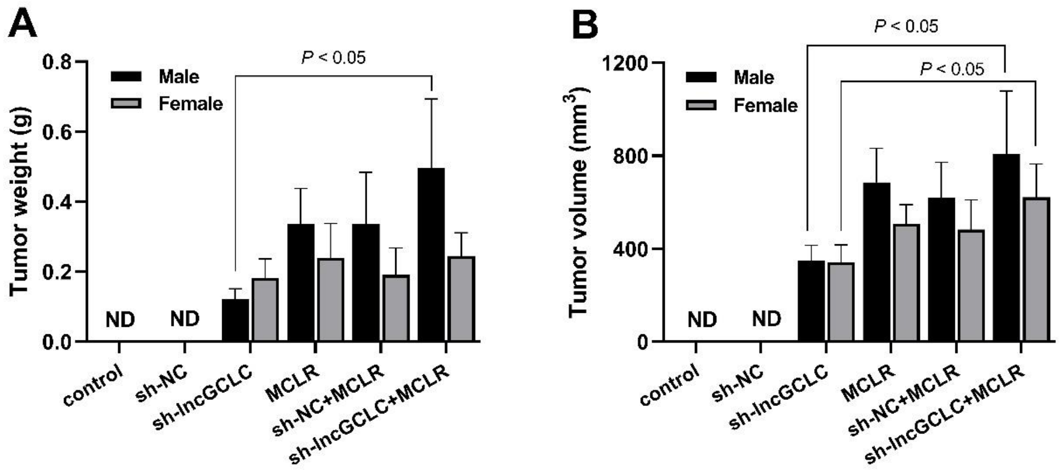

Figure S1. Comparison of tumor weight (A) or volume (B) between male and female nude mice in each group after inoculation for 22 days. ND—not detected. The values given are mean ± SD (n = 3 for both male and female nude mice per group).
